# Supplementary material for: Splice-Junction-Based Mapping of Alternative Isoforms in the Human Proteome
Source: Cell Rep. Author manuscript; Available in PMC 2020 Jan 15. (PMC6961840; doi:10.1016/j.celrep.2019.11.026)
Supplement: 2 [file NIHMS1546469-supplement-2.zip › DF1/PXD009737/TestisLysC/TestisLysC_27_EXOSC10_SDRGFRYNWPQIHQTYLHGRVLSATSATKSDGEMVLP.pdf]

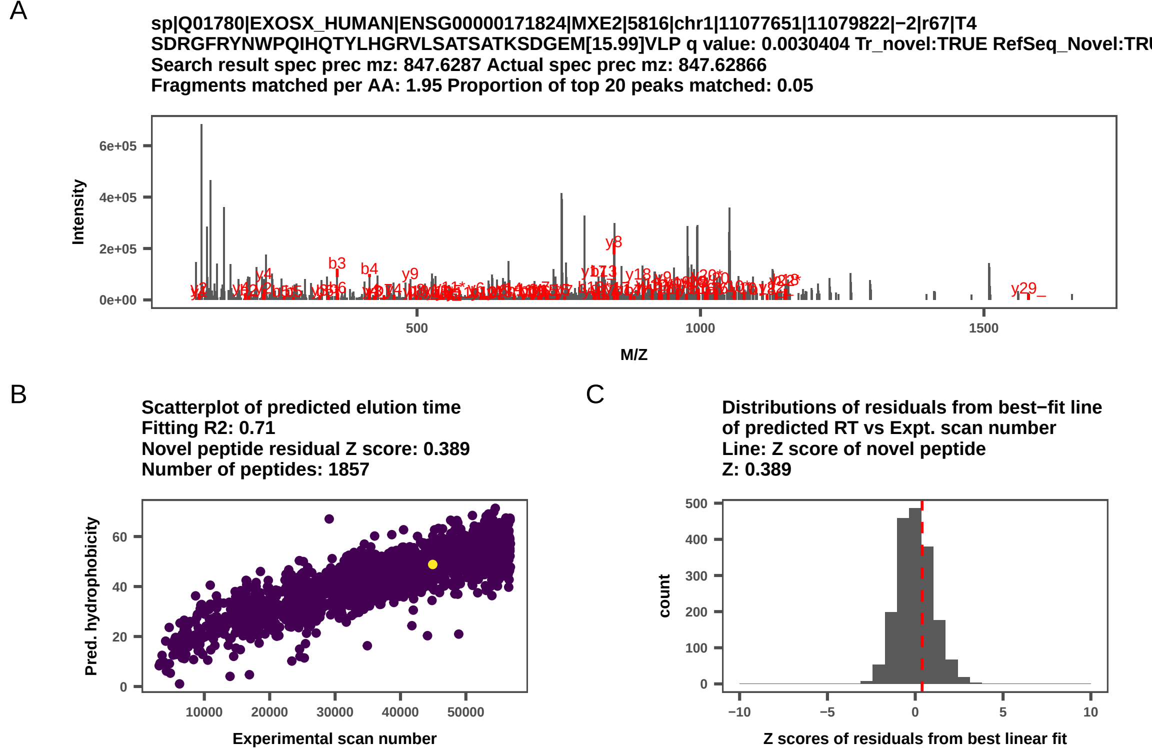
A
sp|Q01780|EXOSX_HUMAN|ENSG00000171824|MXE2|5816|chr1|11077651|11079822|−2|r67|T4
SDRGFRYNWPQIHQTYLHGRVLSATSATKSDGEM[15.99]VLP q value: 0.0030404 Tr_novel:TRUE RefSeq_Novel:TRU
Search result spec prec mz: 847.6287 Actual spec prec mz: 847.62866
Fragments matched per AA: 1.95 Proportion of top 20 peaks matched: 0.05
Intensity
6e+05
4e+05
2e+05
0e+00
y8
b3
b4
y9
y4
y29
500
1000
1500
M/Z
B
Scatterplot of predicted elution time
Fitting R2: 0.71
Novel peptide residual Z score: 0.389
Number of peptides: 1857
Pred. hydrophobicity
60
40
20
0
10000
20000
30000
40000
50000
Experimental scan number
C
Distributions of residuals from best−fit line
of predicted RT vs Expt. scan number
Line: Z score of novel peptide
Z: 0.389
count
500
400
300
200
100
0
−10
−5
0
5
10
Z scores of residuals from best linear fit
